# Remarks from the Editor-in-Chief

**DOI:** 10.1017/psy.2025.10028

**Published:** 2025-07-31

**Authors:** Sandip Sinharay

**Affiliations:** ETS Research Institute

Dear *Psychometrika* Readers,

Welcome to the second Psychometrika issue of 2025. I would like to remind you that Psychometrika is now an Open Access journal, published by Cambridge University Press. Consequently, we are now accepting all submissions of manuscripts (including revisions whose original versions were submitted to the old manuscript submission website) through the new website https://mc.manuscriptcentral.com/psychometrika.

Psychometrika has recently received some makeovers. The section that used to be called the “Application Review and Case Studies” (ARCS) section is now called the “Application and Case Studies” (ACS) section. The former “Book Review” section is being expanded into the “Review” section that will include, instead of only book reviews, reviews of multiple sources of knowledge and tools, including literature, books, software, and media. Thus, literature reviews, which were rarely published in Psychometrika in the past, will be published in the journal going forward. Also, from now, the “Theory and Methods” section will include “focus articles”, which will address topics deemed to be of suitable breadth and significance and will most often be published together with commentaries from peers and a response to the commentaries from the authors of the original article. Focus articles are intended to promote open debate on quantitative methodology in the social and behavioral sciences.

The current issue opens with nine special section articles on the topic *Model Identification and Estimation for Longitudinal Data in Practice*, featured in the “Application and Case Studies” section. In the first of these articles, Michael D. Hunter, Robert M. Kirkpatrick, and Michael C. Neale extend previously published solutions for identifying models with special exogenous variables, known as definition variables, that arbitrarily alter model characteristics. In the second article, Xingyao Xiao, Sophia Rabe-Hesketh, and Anders Skrondal address problematic behaviors of Markov chain Monte Carlo methods for finite mixture models caused by degenerate nonidentifiability. They propose diagnostics to detect such issues and demonstrate that using more informative priors can mitigate these problems in growth mixture models. In the third article, Maarten Speekenbrink and Ingmar Visser, introduces an inhomogeneous hidden Markov model for longitudinal data with state-dependent missingness and describes the model’s estimation using the expectation-maximization algorithm. In the fourth article, Yuanyuan Ji, Jordan Revol, Anna Schouten, Marieke J. Schreuder, and Eva Ceulemans systematically examine the performance of dyadic intensive longitudinal data models in the presence of a large proportion of missing data and suggest alternative models that may be more appropriate when the longitudinal actor–partner interdependence model fails to perform adequately. In the fifth article, Angela Andreella, Jelle Goeman, Jesse Hemerik, and Livio Finos propose a robust extension of the two-stage summary statistics approach to account for within-variance structure and heteroscedasticity, enabling accurate regression coefficient testing in two-level hierarchical data. In the sixth article, Silvia Bianconcini and Kenneth A. Bollen explore how different parameterizations of latent variable structural equation models for binary and ordered categorical repeated measures data can lead to varying conclusions, and they provide closed-form relations between longitudinal model specifications under alternative parameterizations. In the seventh article, Marian M. Strazzeri, Jeffrey R. Harring, and Nan Bernstein Ratner present first-order structured latent curve models for repeated measures count data, where the underlying change process is believed to occur in distinct phases, and discuss issues related to model identification and estimation. In the eighth article, Sun-Joo Cho, Sarah Brown-Schmidt, Sharice Clough, and Melissa C. Duff introduce a model specification using generalized additive mixed models for group comparisons of functional trends over time within a trial, as well as learning across trials, in intensive binary longitudinal eye-tracking data. Finally, in the ninth article, David Kaplan, Kjorte Harra, Jonas Stampka, and Nina Jude demonstrate how optimally predictive growth models can be constructed to monitor the pace at which countries are progressing toward, or diverging from, the education-related Sustainable Development Goals set by the United Nations, and they also discuss Bayesian model identification and estimation issues related to these growth models.

The current issue then includes six “Theory and Methods” section articles. In the first of this set of articles, Jimmy de la Torre, Xuelan Qiu, and Kevin Carl Santos propose the saturated polytomous cognitive diagnosis model (sp-CDM), a general model that subsumes existing CDMs for polytomous attributes as special cases. The second article, written by Luca Stefanutti, Pasquale Anselmi, Debora de Chiusole, Andrea Spoto, and Jürgen Heller, is a rejoinder to a commentary on a 2020 Psychometrika article written by four of these five authors. In the third article, Wen Wu Wang, Xiujin Peng, and Tiejun Tong a novel M-regression approach for mediation analysis that minimizes the Huber loss function with the optimal tuning constant. In the fourth article, Che Cheng, Hau-Hung Yang, and Yung-Fong Hsu examine the identifiability of polychoric models with latent elliptical distributions. The fifth article, by Mariaelena Bottazzi Schenone, Carlo Cavicchia, Maurizio Vichi, and Giorgia Zaccaria, introduces a novel methodology to model the hierarchical dependence structure of manifest variables. The sixth article, by Inhan Kang and Minjeong Jeon, extends the unidimensional Rasch-based latent space item response model by incorporating between-item multidimensionality and item discrimination parameters.

The issue ends with a book review, written by Inés María Varas Cáceres, of the 2025 book “Generalized Kernel Equating with Applications in R” written by Marie Wiberg, Jorge Gonzalez, and Alina A. von Davier.

Hope you enjoy the issue.

